# Treatment effects in multiple cognitive domains in Alzheimer’s disease: a two-year cohort study

**DOI:** 10.1186/alzrt280

**Published:** 2014-08-18

**Authors:** Pearl Behl, Jodi D Edwards, Alexander Kiss, Krista L Lanctot, David L Streiner, Sandra E Black, Donald T Stuss

**Affiliations:** 1 L.C.Campbell Cognitive Neurology Research Unit, Toronto, Canada; 2 University of Toronto, Toronto, Ontario, Canada; 3 Canadian Partnership for Stroke Recovery, Toronto, Ontario, Canada; 4 Brain Sciences Research Program, Sunnybrook Health Sciences Center, Toronto, Ontario, Canada; 5 Department of Psychiatry, Toronto, Ontario, Canada; 6 Department of Psychiatry & Behavioral Neurosciences, McMaster University, Toronto, Ontario, Canada; 7 Department of Medicine (Neurology), Toronto, Ontario, Canada; 8 Department of Psychology, Toronto, Ontario, Canada; 9 Ontario Brain Institute, Toronto, Ontario, Canada

## Abstract

**Introduction:**

Despite widespread use of second-generation cholinesterase inhibitors for the symptomatic treatment of Alzheimer’s disease (AD), little is known about the long term effects of cholinergic treatment on global cognitive function and potential specific effects in different cognitive domains. The objectives of this study were to determine the association between cholinergic treatment and global cognitive function over one and two years in a cohort of patients with mild or moderate AD and identify potential differences in domain-specific cognitive outcomes within this cohort.

**Methods:**

A cohort of patients meeting the revised National Institute of Neurological and Communicative Disorders and Stroke and the Alzheimer's Disease and Related Disorders Association (NINCDS-ADRDA) criteria for mild or moderate AD, including patients both on treatment with a cholinesterase inhibitor and untreated controls (treated = 65, untreated = 65), were recruited from the Cognitive Neurology Clinic at Sunnybrook Health Sciences Centre, as part of the Sunnybrook Dementia Study. Patients were followed for one to two years and underwent standardized neuropsychological assessments to evaluate global and domain-specific cognitive function. Associations between cholinesterase inhibitor use and global and domain-specific cognitive outcome measures at one and two years of follow-up were estimated using mixed model linear regression, adjusting for age, education, and baseline mini mental state examination (MMSE).

**Results:**

At one year, treated patients showed significantly less decline in global cognitive function, and treatment and time effects across tests of executive and visuospatial function. At two years, there was a significant trend towards less decline in global cognition for treated patients. Moreover, treated patients showed significant treatment and time effects across tests of executive functioning, memory, and visuospatial function.

**Conclusions:**

The present study offers two important contributions to knowledge of the effectiveness of cholinesterase inhibitor treatment in patients with mild-moderate AD: 1) that second-generation cholinesterase inhibitors demonstrate long-term effectiveness for reducing global cognitive decline over one to two years of follow-up, and 2) that decline in function for cognitive domains, including executive function, memory, and visuospatial skill that are primarily mediated by frontal networks and by the cholinergic system, rather than memory, may be slowed by treatment targeting the cholinergic system.

## Introduction

Alzheimer’s disease (AD) affects more than 35 million people worldwide [1,2] and is the most common form of dementia in older people. Memory impairment is one of the essential and earliest manifestations of AD [3-5], and accompanying deficits include difficulties with word finding [2], visuospatial [6] and executive function impairment [7].

AD is classified as one of the cortical dementias and, although the etiopathogenesis of the disease remains undefined, deficits in memory and cognition have previously been associated with cholinergic deficits in both the hippocampus and cerebral cortex [8]. Randomized double-blind, placebo-controlled trials of three second-generation cholinesterase inhibitors (ChEIs) – donepezil, rivastigmine, and galantamine – that enhance synaptic concentrations of acetylcholine have demonstrated modest beneficial treatment effects in mild to moderate AD over 6 to 12 month periods [9-13]. However, two major gaps regarding treatment targeting the cholinergic system that have previously not been addressed in randomized trials relate to the short duration of follow-up for treatment effects and the lack of information on specific cognitive domains.

Most clinical trials have only examined patients over shorter term periods of follow-up, so the duration of treatment effects have not been well characterized [14,15]. The reasons for this are related primarily to the disease process itself: it is difficult to conduct symptomatic treatment studies in a relentlessly progressive neurodegenerative disorder such as AD over time periods longer than 6 months within the context of a clinical trial because, given the efficacy shown with ChEIs, longer term placebo groups may no longer be considered ethical. Longer follow-up periods of up to 18 months are being pursued in trials with disease-modifying potential, with the experimental treatment being added on to stable approved symptomatic therapy.

Another issue has been whether treatment benefits are disabled in trials of longer duration. Four industry-sponsored, double-blind, placebo-controlled trials have investigated the efficacy of continued ChEI treatment over 1 year [16-18] and 2 years [19] of follow-up. These studies demonstrated significant benefits, but only global cognition was evaluated in treated patients [16,17]. A nonpharmaceutical-sponsored, randomized, double-blind trial investigating the effects of donepezil over 2 years also revealed less decline in overall cognition and activities of daily living in mild to moderate AD associated with treatment [19]. However, this trial was limited by a large dropout rate for patients (40% at the end of 1 year and 77% at the end of 2 years).

The interpretation of available data from even longer term trials is difficult, since these are open-label extensions [20] also confounded by large dropout rates and the bias that comes from patient self-selection. A natural history extrapolation using the Stern equation is typically used for comparison instead of real data [21]. More recently, ChEIs have been the open-label comparator for some newer putative disease-modifying therapy trials, which include untreated patients who provide a true placebo comparison but are a small subset of the overall trial population [22,23]. Most studies use the last observation carried forward, a flawed approach when the objective is to investigate slower progression and decline [24]. One possible method for obtaining important longer term outcome information is to employ well-conducted longitudinal cohort studies [25].

A second limitation of previous studies has been the emphasis on global cognitive outcome measures, with little information on domain-specific cognitive outcomes. Separate dissociable neuropsychological impairments have been shown in AD, yet it is unknown whether attention, executive, memory, visuospatial, and language functions are all equally likely to benefit from a particular pharmacotherapy, and there are currently no randomized controlled trial data assessing treatment effects on performance for specific cognitive domains. Of particular importance are executive functions, which have previously been correlated with instrumental activities of daily living [26] and may be particularly responsive to cholinergic agents, given the known effects of the cholinergic system on selective attention [27,28]. Preliminary findings in a previous study from our group indicated that executive, language and visuospatial functions, as indexed by the Mattis Dementia Rating Scale (DRS) [29] and its five subscores, rather than memory, appeared more amenable to stabilization by ChEIs in AD over 1 year [30]. Given the natural course of AD degeneration over several years, it is essential to assess the potential benefits of ChEIs over time periods longer than 6 months to evaluate whether these drugs have lasting effects in AD patients. Of equal importance is the assessment of treatment benefits for different cognitive domains, to identify potential domain-specific differences in treatment response in this clinical population.

The present study sought to address these gaps in the knowledge of the effects of cholinesterase therapy in mild or moderate AD. The primary objective was to examine the association between treatment with ChEIs and global cognitive function over 1 year and 2 years of follow-up in mild or moderate AD patients. The secondary objective was to evaluate domain-specific cognitive performance over this longer term follow-up period, to determine whether specific domains are differentially affected in mild or moderate AD patients treated with ChEIs compared with untreated patients.

## Methods

### Cohort selection

One-hundred and thirty patients (untreated = 65, treated = 65) meeting the revised National Institute of Neurological and Communicative Disorders and Stroke–Alzheimer's Disease and Related Disorders Association criteria for mild (baseline Mini-Mental Status Examination (MMSE) 20–30) or moderate (baseline MMSE 10–19) probable AD [31] were recruited from the Cognitive Neurology Memory clinic at Sunnybrook Health Sciences Centre, a University of Toronto academic healthcare institution, for the period from 1993 to 2002. Inclusion and exclusion criteria for the study cohort have been described previously [30,32]. This study had ethical approval from the Sunnybrook Research Ethics Board (REB PIN: 009–1998) and all patients selected for the study cohort provided informed consent prior to participation. Details regarding selection procedures for the final cohort have also been described in previous publications [30,32].

### Study variables and procedures

All patients underwent a standardized neuropsychological protocol at baseline and 1 year of follow-up, with a subset of patients also assessed at a 2-year follow-up session. The primary outcome was performance on standardized measures of global cognitive function, described below. Secondary outcomes included performance on multiple measures of domain-specific cognitive function.

Demographic variables included: age; sex; education, which was categorized as elementary (0 to 6 years), high school (7 to 12 years), and post-secondary (≥13 years); and baseline score on the MMSE, categorized into mild (≥20) and moderate (10 to 19) categories. To minimize the potential impact of bias due to losses to follow-up, baseline demographics of all patients diagnosed with mild or moderate probable AD who began treatment between 1997 and 2002 were examined and lost observations were compared with those who stayed on therapy and were followed to the study endpoint. To minimize the potential for bias associated with the use of a historical control cohort, data on potential confounding variables including use of concomitant medications, presence of comorbid illnesses, vascular risk factors (hypertension, diabetes mellitus, hyperlipidemia, smoking), family history of stroke, and the presence of vascular end-organ damage were prospectively ascertained for all patients and validated by a second review of clinic charts [30]. Mean depressive symptomatology scores from the Cornell Scale for Depression in Dementia [33] for were obtained for all patients and concomitant use of selective serotonin reuptake inhibitors was carefully documented.

### Treatment

All of the patients included in the study cohort were enrolled in a longitudinal observational study, the Sunnybrook Dementia Study [ClinicalTrials.gov:NCT01800214], using the same standardized protocol either prior to the approval of ChEIs (no-treatment cohort) or after treatment (treatment cohort) became a common clinical option. Once cholinergic therapy was available, all patients with mild to moderate probable AD were offered treatment, if not contraindicated, and titrated as recommended to a maximum tolerated dose (achieved in >90% of patients in the study).

### Neuropsychological assessment protocol

#### Global cognition

Global cognitive function was assessed using the MMSE [34] and the total score from the DRS [29].

#### Domain-specific cognition

Memory function was assessed using the California Verbal Learning Test (acquisition, and short and long delay free recall subscores) [35], delayed visual reproduction [36], and the memory subscore of the DRS [29]. The Backward Digit Span task was included as a measure of working memory function [36]. Executive functioning was indexed with several measures, including the Controlled Word Association Test using the letters F, A and S (phonemic fluency, correct) [37], Semantic Fluency for animals (words correct) [37], Wisconsin Card Sort Test (number correct, denoting the number of categories correctly completed) [38], Trails B (time in seconds) [39], and the attention, conceptualization and initiation/perseveration subscores of the DRS [29]. Trails A (time in seconds) was used to measure speed of processing. Language function was assessed using the Boston Naming Test (30-word version) [40]. Visuospatial attention and visuoconstructive skill were measured with the Rey–Osterreith Complex Figure Test [41], the Benton Line Orientation Test [42], and the construction subscore of the DRS [29].

### Statistical analyses

Paired-sample *t* tests and chi-square analyses were used to compare treated and untreated patients with respect to all continuous and categorical demographic variables at baseline (see Table 1). Critical values for significance were corrected for multiple comparisons using the Holm correction [43], a sequentially rejective Bonferroni method to maintain the experiment-wise error rate.

**Table 1:** **Baseline characteristics for treated (*n*** **= 65) and untreated (*n*** **= 65) patients with mild–moderate Alzheimer’s disease**

**Factor**	**Untreated**	**Treated**	**χ**^**2 **^**/ *t *** **statistic (df)**	***P *** **value**^**a**^
Males/females	32/33	36/29	0.50 (1)	0.50
Age	71.4 ± 8.4	71.9 ± 9.9	0.70 (128)	0.70
Education (years)	13.5 ± 3.5	13.3 ± 3.9	0.50 (128)	0.50
Handedness			0.40 (1)	0.80
Right	60	63		
Both	5	2		
Handedness _2_			0.021 (1)	0.90
Right	32	55		
Both	2	3		
Symptom duration (years)	3.5 ± 2.3	2.8 ± 2.3	0.11 (128)	0.80
Mini-Mental State Examination	22.4 ± 3.7	23.1 ± 3.6	1.1 (128)	0.30
Dementia Rating Scale	116.8 ± 11.0	118.8 ± 14.3	0.98 (128)	0.32
Disability Assessment for Dementia	78.9 ± 15.9	82.8 ± 17.3	0.10 (128)	0.90
Instrumental activities of daily living	65.9 ± 25.2	72.2 ± 24.6	0.10 (128)	0.92
Basic activities of daily living	95.8 ± 5.9	95.2 ± 9.1	1.90 (128)	0.06
BEHAVE-AD	5.3 ± 3.6	5.6 ± 6.5	0.20 (128)	0.80
Hypertension	24	16	2.3	0.10
Hyperlipidemia	9	11		0.80
Diabetes	4	3		1.00
Smoking			7.4	0.02
Never	42	32		
Current	6	2		
Previous	17	31		
Family history of stroke	10	13		0.65
Cerebrovascular disease	4	8		0.36
Cardiac disease	11	8		0.60
Peripheral vascular disease	5	1		0.21
Chronic respiratory, gastrointestinal and genitourinary disease	36	47	1.90	0.20
Depressive symptoms (not due to primary psychiatric illness)	0	3		0.20
Neurological problems (for example, migraines)	8	14		0.20
Endocrine/metabolic deficiency/infectious diseases	10	7		0.60
Chemical exposure	4	1		0.40
Ear–nose–throat	15	16		1.00
Cancer	7	9		0.80
Musculoskeletal disease	19	25	1.2	0.27
Statins	8	7		1.00
Selective serotonin reuptake inhibitors	9	17		0.12
Cardiovascular	23	19	0.56	0.45
Antithrombotics	22	22	0.0	1.00
Non-steroidal anti inflammatory drugs	4	2		0.68
Antipsychotics	1	0		1.00
Anticonvulsants	3	5		0.72
Diabetes	3	2		1.00
Hormone replacement therapy	8	9		1.00
Thyroid	9	8		1.00

Data presented ± standard deviation unless otherwise stated. Fisher’s exact test was used for cells with counts ≤15. BEHAVE-AD, Behavioural Pathology in Alzheimer's Disease; df, degrees of freedom. ^a^Critical values corrected for multiple comparisons (Holm’s correction) – no significant difference.

Treatment effect sizes (Cohen’s *d*) were calculated as standardized response means for all patients at both the 1-year and 2-year follow-up endpoints:

$$\frac{\left({T_{t2‒}}_{t1}-{U_{t2‒}}_{t1}\right)}{{\left(\mathrm{SD}\right)}_{\mathrm{pooled}}}$$

where **T** is the treated group, **U** is the untreated group, t2 is the follow-up, t1 is the baseline, and SD_pooled_ are the standard deviations of the change scores for the two groups (Cohen’s criteria: 0.2 = small effect size, 0.5 = moderate effect size, 0.8 = large effect size).

Associations between treatment with ChEIs and cognitive outcome measures were estimated using multivariable mixed-model linear regression, with separate models derived for all cognitive outcomes at 1 year and 2 years of follow-up. All final models were adjusted for age, education, and baseline MMSE score.

## Results

Mean follow-up times for the 1-year assessment were 14.4 months for untreated patients and 14.6 months for treated patients. Mean follow-up times for the 2-year assessment were 25.2 months for untreated patients and 26 months for treated patients. There were no significant differences in mean follow-up between treated patients and untreated patients at either time point.

Baseline demographics and characteristics are provided in Table 1. No significant differences were observed between treated patients and untreated patients for baseline demographic variables, baseline neuropsychological performance, nonvascular and vascular disease burden indices at baseline or at follow-up, body system or chemical exposure, alcohol use, allowable neurological problems (such as history of migraine), or remote history of depressive symptoms. Patients also did not differ in terms of cerebrovascular, cardiac, or peripheral vascular disease prevalence, or in use of concomitant medications including statins, antihypertensives and hypoglycemic agents at baseline or follow-up (see Table 1).

### Primary outcomes

**Figure 1:**
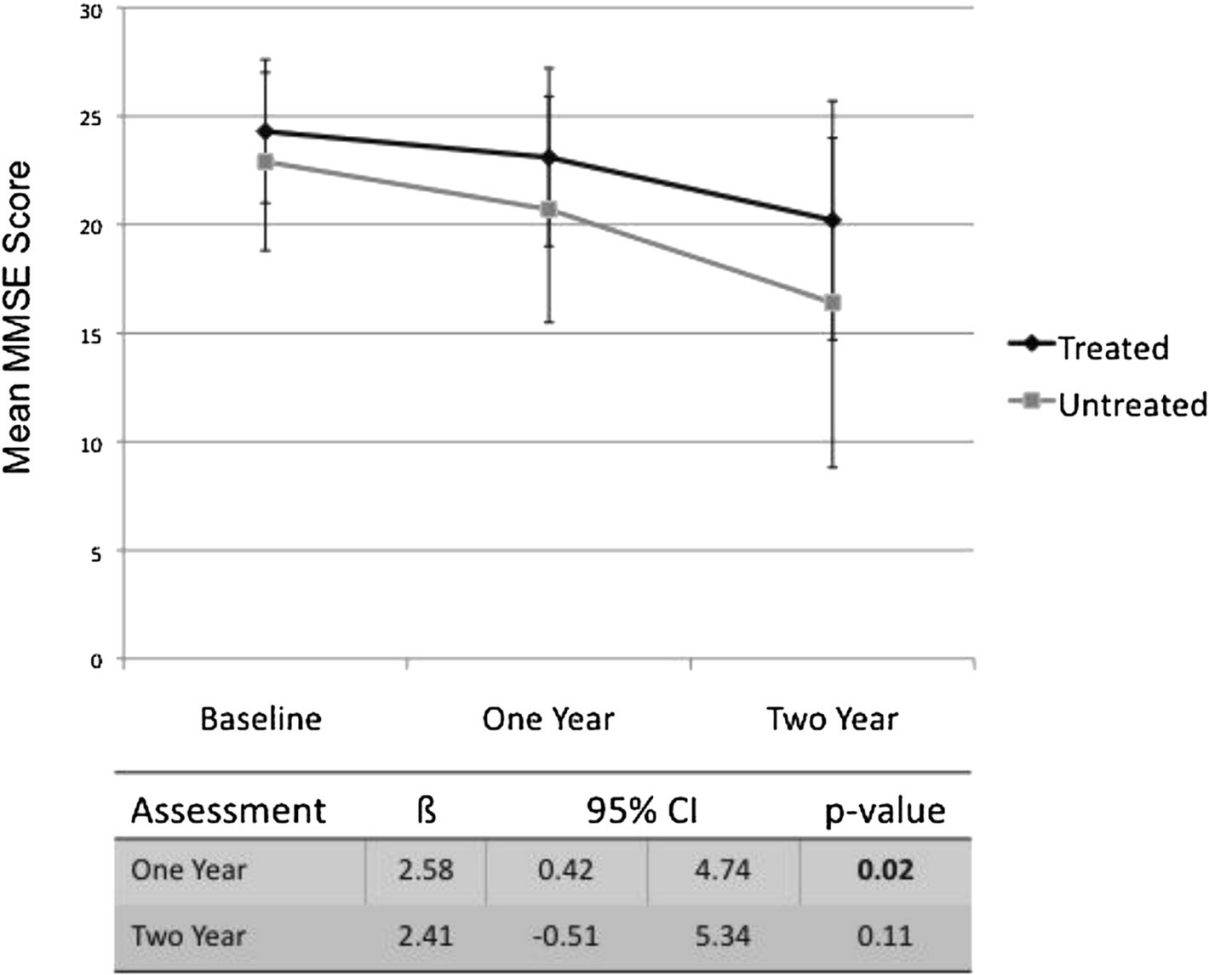
**Change in mean Mini-Mental Status Examination over time and estimate of treatment effect for treated and untreated patients with mild–moderate Alzheimer’s disease.** CI, confidence interval; MMSE, Mini-Mental Status Examination.

**Figure 2:**
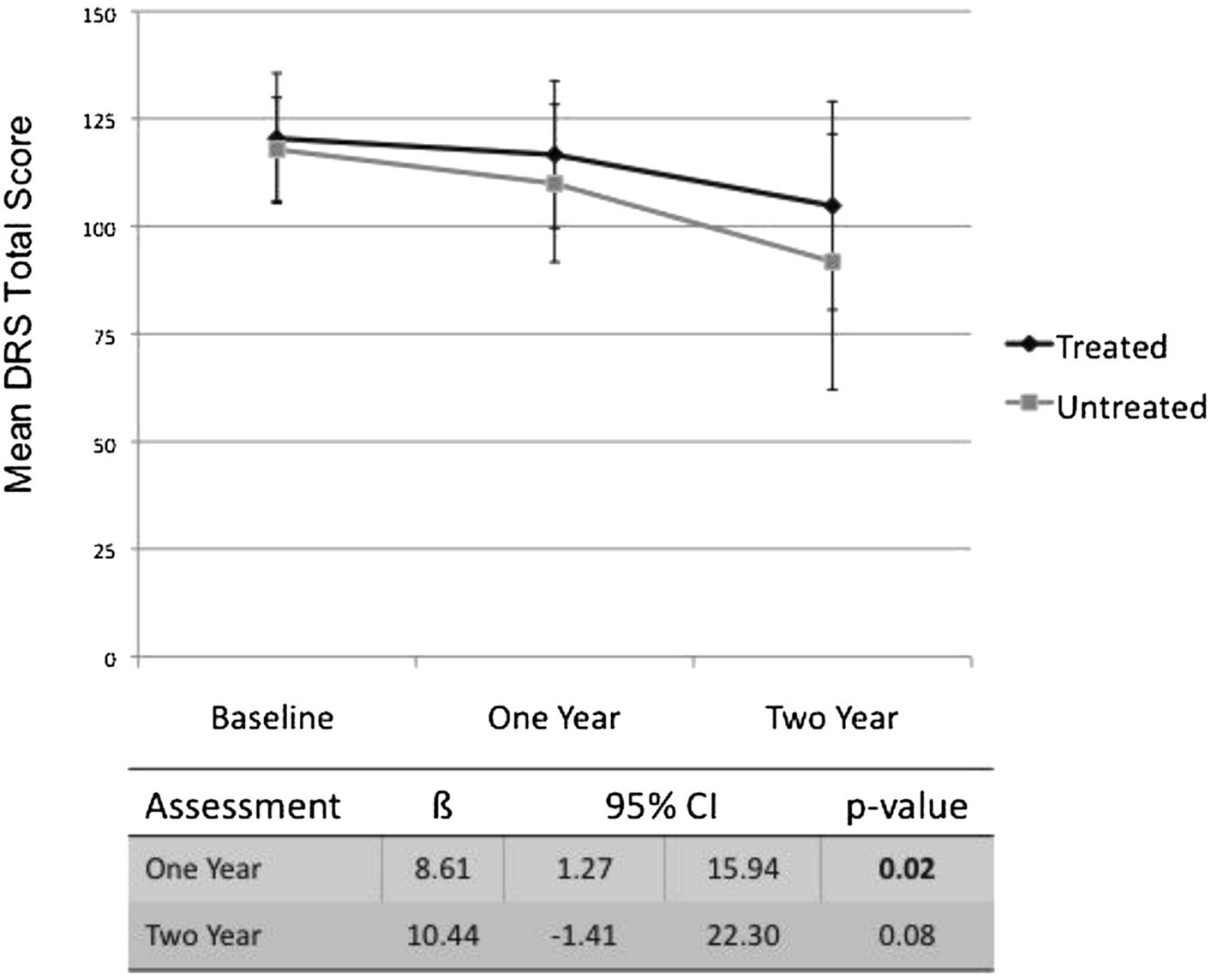
**Change in mean Dementia Rating Scale total score over time and estimate of treatment effect for treated and untreated patients with mild–moderate Alzheimer’s disease.** CI, confidence interval; DRS, Dementia Rating Scale.

Mean scores on the MMSE and the DRS total for treated patients and untreated patients at baseline and at the 1-year and two-year assessments, and effect size estimates, are presented in Table 2. Results of mixed linear regression modeling demonstrated significant differences in global cognitive function over time between treated patients and untreated patients. Specifically, treated patients showed significantly less decline from baseline to the 1-year assessment than untreated patients on both the MMSE (interaction: ß = 2.58, 95% confidence interval (CI) = 0.42 to 4.74, *P* = 0.02) (see Figure 1) and the DRS total score (interaction: ß = 8.61, 95% CI = 1.27 to 15.94, *P* = 0.02) (see Figure 2), after adjusting for age and educational status. Although significant differences in MMSE score were also observed between baseline and the 2-year assessment across treatment status (main effect: ß = 26.14, 95% CI = 16.85 to 35.44, *P* < 0.01), differences between treated patients and untreated patients over time on this measure did not reach significance in the adjusted model (interaction: *P =* 0.11). Similarly, significant differences were also observed between baseline and the 2-year assessment across treatment status for the DRS total score (main effect: ß = 6.53, 95% CI = 4.23-8.82, *P* < 0.01), but these differences only trended towards significance by treatment status in the adjusted model (interaction: *P* = 0.08) (Figures 1 and 2).

**Table 2:** Mean (standard deviation) and effect size estimates for global measures of cognitive function over time in treated and untreated patients with mild–moderate Alzheimer’s disease

**Measure**	**Assessment time**	**Mean (standard deviation)**		**Effect size**
		**Treated**	**Untreated**	**(Cohen’s *d*)**
MMSE	Baseline	24.3 (3.3)	22.9 (4.1)	
	1 year	23.1 (4.1)	20.7 (5.2)	0.7
	2 years	20.2 (5.5)	16.4 (7.6)	0.5
DRS total	Baseline	120.5 (15.1)	117.9 (12.0)	
	1 year	116.7 (17.1)	110.0 (18.3)	0.7
	2 years	104.8 (24.2)	91.8 (29.7)	0.5

DRS, Dementia Rating Scale; MMSE, Mini-Mental State Examination.

### Secondary outcomes

Mean scores on all domain-specific cognitive measures for treated and untreated patients at baseline and at the 1-year and 2-year assessments, and effect size estimates, are presented in Table 3. Owing to a high proportion of missing observations (reflecting task difficulty), data for the California Verbal Learning Test and Trails A and Trails B measures were not included in these analyses.

**Table 3:** Mean (standard deviation) and effect size estimates for domain-specific measures of cognitive function over time in treated and untreated patients with mild–moderate Alzheimer’s disease

**Measure**	**Assessment**	**Mean (standard deviation)**		**Effect size**
	**Time**	**Treated**	**Untreated**	**(Cohen’s *d*)**
DRS attention	Baseline	34.5 (3.2)	35.1 (2.0)	
	1 year	33.6 (3.9)	33.6 (3.9)	0.50
	2 years	31.7 (5.2)	30.2 (7.8)	0.40
DRS initiation	Baseline	29.6 (6.1)	27.2 (6.2)	
	1 year	28.6 (6.5)	25.4 (7.8)	0.51
	2 years	24.6 (9.2)	19.6 (8.7)	0.50
DRS construction	Baseline	5.0 (1.2)	5.4 (1.4)	
	1 year	5.0 (1.3)	4.9 (1.8)	0.52
	2 years	4.3 (1.7)	4.4 (2.0)	
DRS conceptualization	Baseline	33.3 (5.8)	34.2 (3.8)	
	1 year	34.4 (4.6)	32.0 (6.3)	0.53
	2 years	31.4 (7.1)	27.2 (10.5)	0.60
DRS memory	Baseline	16.9 (4.0)	15.8 (4.3)	
	1 year	15.5 (4.2)	14.1 (4.0)	
	2 years	12.3 (5.0)	10.4 (4.1)	
VR2	Baseline	3.4 (5.9)	4.4 (5.6)	
	1 year	3.0 (5.4)	1.6 (3.8)	
	2 years	1.7 (5.4)	1.3 (2.6)	
BDS	Baseline	5.3 (2.2)	5.1 (2.5)	
	1 year	4.9 (2.1)	4.5 (2.4)	0.40
	2 years	4.9 (2.5)	3.9 (2.5)	0.50
REY	Baseline	25.7 (9.0)	25.5 (10.8)	
	1 year	25.1 (10.1)	21.4 (13.8)	0.5
	2 years	22.1 (10.6)	17.2 (24.2)	
Benton Line Orientation Test	Baseline	20.6 (7.6)	19.8 (9.1)	
	1 year	18.8 (9.8)	17.2 (9.7)	
	2 years	17.7 (9.5)	16.1 (10.3)	
WCST	Baseline	41.5 (8.3)	36.9 (7.7)	
	1 year	41.3 (8.8)	35.3 (10.3)	
	2 years	36.6 (9.3)	32.6 (10.1)	
Boston Naming Test	Baseline	22.2 (5.4)	20.9 (6.3)	
	1 year	20.3 (6.7)	17.5 (7.5)	0.50
	2 years	18.7 (8.3)	13.3 (8.5)	0.60
Semantic Fluency	Baseline	12.4 (4.4)	10.1 (4.3)	
	1 year	10.6 (5.1)	7.9 (4.2)	
	2 years	8.9 (4.2)	5.8 (4.0)	
FAS	Baseline	31.6 (12.7)	24.8 (11.6)	
	1 year	30.3 (11.6)	21.3 (13.2)	0.73
	2 years	24.0 (11.8)	17.6 (12.1)	

BDS, Backward Digit Span; DRS, Dementia Rating Scale; FAS Controlled Word Association Test; REY, Rey–Osterreith Complex Figure Test; VR2, delayed visual reproduction; WCST Wisconsin Card Sort Test.

Overall, results for the secondary outcomes at the 1-year follow-up assessment showed significant differences between treated patients and untreated patients across time-points on measures of executive function and visuospatial attention and visuoconstructive skill, and significant differences between baseline and 1-year follow-up across treatment status on measures of executive function; however, no significant interactions between treatment and time at 1 year were present in the adjusted models for any of the domain-specific cognitive measures. Specifically, significant differences between treated patients and untreated patients were observed across time from baseline to 1 year on the Wisconsin Card Sorting Test (main effect: ß = 3.76, 95% CI = 0.03 to 7.49, *P* = 0.05) and the Benton Line Orientation Test (main effect: ß = 3.88, 95% CI = 0.01 to 7.78, *P* = 0.05). Significant differences from baseline to 1-year follow-up across treatment status were also observed for the Semantic Fluency task (main effect: ß = 1.49. 95% CI = 0.02 to 2.95, *P* = 0.04).

At the 2-year follow-up assessment, results showed a significant difference between baseline and the 2-year assessment across treatment status on a measure of executive function, and trends towards significance by treatment and by time for several domains, including executive function, memory, visuospatial attention, and visuoconstructive skill. Specifically, significant differences between treated patients and untreated patients were observed across time from baseline to 2 years on the Semantic Fluency test (main effect: ß = 1.78, 95% CI = 0.23 to 3.34, *P* = 0.02). Trends towards significant main effects for treatment status across time were observed for the attention (*P =* 0.08) and construction (*P* = 0.06) subscores of the DRS, the Controlled Word Association Test (*P* = 0.06), and the Semantic Fluency test (*P =* 0.09). Trends towards significant main effects for time across treatment status were observed for the attention (*P =* 0.10) and memory (*P =* 0.07) subscores of the DRS, and for the Controlled Word Association Test (*P =* 0.10). No significant interactions between treatment and time at 2 years and no significant main effects were present in the adjusted models for any of the other domain-specific cognitive measures.

## Discussion

The purposes of this study were to evaluate the effect of treatment with a ChEI on long-term global and domain-specific cognitive performance in patients with mild to moderate AD. To our knowledge, this observational cohort study represents the first to assess longer-term cognitive performance across multiple cognitive domains using a detailed, standardized neuropsychological battery for each of these treatment groups. Findings of this study indicated that treated patients with mild or moderate AD showed slower decline in global cognitive function over 1 year and a trend towards slower decline over 2 years, as compared with untreated AD patients. In addition, secondary analyses indicated that performance on tasks requiring the domains of executive function, memory, visuospatial attention, and visuoconstructive skill differed either between groups or over time in this cohort, suggesting that, in addition to memory, other specific cognitive domains may be selectively responsive to long-term cholinesterase treatment in mild to moderate AD patients. The present study thus offers two important contributions to knowledge of the effectiveness of ChEI treatment in this clinical population: second-generation ChEIs demonstrate long-term effectiveness for reducing global cognitive decline over 1 to 2 years of follow-up; and decline in function for several cognitive domains, including executive function, memory, and visuospatial skill, may be slowed by treatment targeting the cholinesterase system.

The findings of the present study are consistent with data from previous 6-month pivotal clinical trials and provide further evidence to support the benefit of ChEI treatment for global cognitive function in patients with mild–moderate AD. Although the short-term effectiveness of second-generation ChEIs has been demonstrated previously in double-blind, placebo-controlled trials, few data were available evaluating the effectiveness of ChEIs over 1 and 2 years. In comparison with untreated AD patients, treated patients in the current study demonstrated significantly less decline in global cognitive function, as measured using the MMSE and DRS, over a 1-year period and trends towards less decline over 2 years. Further, these benefits exhibited moderate effect sizes, greater in many instances than those of the Alzheimer’s Disease Assessment Scale – cognitive subscale differences in the 6-month pivotal trials [44], and were present for the DRS, which is a more sensitive measure of cognitive progression across a broader disease spectrum in patients with AD [45]. These findings have implications for the use of treatment targeting the cholinesterase system in patients with mild to moderate AD, and suggest that these patients may benefit from long-term treatment with ChEIs to slow the progression of cognitive impairment.

Several factors support the validity and comparability of our patients with previous randomized clinical trial populations. Our patients were similar with respect to average age and education, but had a slightly higher mean MMSE scores at baseline than the pivotal trials. The literature shows an average annual decline in untreated AD patients of 2 to 4 points for the MMSE [46]. Our data showed a decline of 3.6 points over a 1-year period in the untreated patients. This is also comparable with the untreated controls in the cohort of Lopez and colleagues, who showed a 4-point decline over 1 year [25]. Furthermore, our study showed a decline of 0.9 points on the MMSE in the treated group, which is also comparable with the year-long, placebo-controlled, randomized clinical trials that ranged from an improvement of 1 point to a decline of 2 points in the treated patients depending on baseline disease severity [16-19,25,47]. The results of the present study are hence consistent with previous data from clinical trials with respect to the mean change in MMSE score and suggest that trends toward slower decline in treated patients may also persist up to 2 years of follow-up.

The present study also extends findings of previous double-blind, randomized, placebo-controlled trials [17-19] by providing information to characterize potential differences in treatment effects for specific cognitive domains. Unlike prior work, analyses of secondary outcomes in the present study indicated that the cognitive domain primarily responsive to treatment in AD patients was executive function, not memory, with significant main effects observed for both treatment status and time on several measures of executive function. *In vivo* studies using structural brain imaging have shown significant correlations between severity of hippocampal damage and delayed recall measures on the California Verbal Learning Test [48] and the memory subscale of the DRS [49]. The early isolation of the hippocampus from other regions and the resulting loss of basal forebrain cholinergic projections are thought to be key neural substrates for the severe memory deficits observed in AD. Thus, increasing synaptic concentrations of acetylcholine may be less effective in medial temporal cortices, which are selectively vulnerable to AD pathology and may already demonstrate major pathology by the time of clinical symptom onset. This is consistent with findings from our study, as treated patients did not show beneficial differences on tasks of memory function, including the DRS memory subscore, as compared with untreated patients over 1 and 2 years.

Cholinergic augmentation may have a greater impact on the attentional system [7,50], such that tasks measuring attention and executive dysfunction may show greater sensitivity to ChEI treatment effects in AD patients than those that measure memory. There is increasing evidence from clinical studies to support this possibility. Previous studies examining the effects of the cholinergic agonist tacrine reported benefits in a five-choice attention task in AD patients with improvements in speed and accuracy, but not memory [51,52]. Further, data from one small randomized controlled trial comparing 15 treated AD patients with 20 placebo-treated patients showed less decline for treated patients on the Backward Digit Span over 1 year [53], and a previous Japanese cohort study comparing 47 AD patients treated with ChEIs with 61 untreated patients showed less decline in treated patients in concept formation and abstract thinking skills over 10 months [54].

In the present study, treated patients and untreated patients differed significantly at baseline and 1 year in performance on the Wisconsin Card Sorting Test, providing further support for the benefit of ChEI treatment for executive functions involving selective strategic planning and organization, goal-directed behavior, and cognitive shift setting. In addition, trends towards significant differences by treatment status were also present for the attentional and constructional subscores of the DRS at 2 years, suggesting the potential for long-term cholinesterase therapy to stabilize deficits in the ability to attend to and execute verbal and visual commands of varied complexity. These findings are consistent with prior literature demonstrating the efficacy of ChEI treatment for attentional and executive functions, and provide further evidence to suggest that, given the facilitative effects of the cholinergic system on attention, long-term treatment with ChEIs may offer benefits for executive functioning in patients with mild–moderate AD [55-63].

Visual–spatial and visuoconstructional impairment in AD are also attributed to impaired executive functioning [64,65]. Stabilization of performance on visuoconstructive tasks, such as the Rey copy task, has previously been shown in a small clinical study of 15 AD patients treated with a ChEI compared with 20 placebo-treated control patients [53]. Although results of the present study showed no differences between patients on performance for the Rey copy task, a significant main effect of treatment status was observed at 1 year for the Benton Line Orientation Test, another task involving visual–spatial and visuoconstructive skill, suggesting that these networks may also be selectively responsive to treatment targeting the cholinergic system.

Tasks of verbal fluency provide a measure of encoding deficits associated with an inability to utilize semantic knowledge to characterize words and assist in learning [66-68] characteristic of early AD [69-71]. For instance, hippocampal atrophy has previously been correlated with recall on a list-learning task and has been associated with reduced memory-enhancing effects of ChEIs [52]. Similarly, in a recent study using the CERAD word list learning test, (The Consortium to Establish a Registry for Alzheimer's Disease) 14 AD patients treated with ChEIs showed no difference in delayed free recall compared with 14 untreated patients, but treated patients made fewer errors on a recognition test [72]. Results from our study showed significant main effects by both treatment status and time at 1 and 2 years on the semantic fluency task and an additional trend for main effects of treatment and time at 2 years on the Controlled Word Association Test, a task of phonemic fluency. These findings indicate that, despite the potential for hippocampal pathology in patients with mild–moderate AD, benefits may still be provided by ChEI treatment for the strategic processes involved in verbal fluency [73-77].

Although randomized controlled trials are considered the gold standard for comparing the efficacy of cholinesterase therapies in patients with neurodegenerative conditions, such as AD, these trials are limited by ethical and practical issues with randomization. Observational cohort studies provide an alternative methodology for the investigation of long-term treatment effects in this clinical population. The present study followed treated patients and untreated patients within a longitudinal cohort for up to 2 years and also explored domain-specific cognitive functioning, minimizing the limitations of longer randomized controlled trials (ethics of a placebo group; significant dropout). Despite these strengths, the present study also had limitations. Losses to follow-up were a factor in the present study, limiting our ability to explore the effects of treatment for some cognitive measures. However, the use of mixed-model analytic techniques minimized the impact of these losses and enabled us to examine function across several cognitive domains. It is also important to note that the use of historical control groups is subject to biases, such as selection bias and the Flynn effect, which may have impacted these analyses. Although the Flynn effect can influence the comparison of cohorts from different time periods, the potential for this bias was minimal in the present study, as the time gap between intake procedures for patients selected for this cohort was very short (1993 to 1996 for untreated patients and 1997 to 2002 for treated patients). The short time gap for cohort selection also reduced potential biases due to changes in the prevalence of comorbid conditions that may have occurred over the study period. Further, the potential for selection biases were also minimized in the present study, via the ascertainment, validation, and comparison of groups within the study cohort on several potential confounding factors. However, it is possible that, despite these methods, residual biases associated with the use of a historical control remained.

## Conclusions

Findings from our study provide evidence that treatment with ChEI offers significant long-term benefits for global cognitive function in patients with mild to moderate AD. Observations of stabilization in performance on tasks of executive, attentional, visuospatial, and visuoconstructive functions [78-80] across time and treatment also suggest that ChEIs may selectively preserve function for these specific cognitive domains. It is important to note that executive functions, mediated by frontal regions, rather than memory, showed greater potential response to ChEI in this cohort. These findings have implications for the identification of regions with potential differential capacities for response to cholinergic therapies. As efforts to identify disease-modifying treatments with the ability to slow or interrupt early pathologic changes, prevent disease progression, and alter the natural course and outcome of AD have been unsuccessful to date [23], further research is required to optimize therapeutic strategies for patients with AD. This study offers important insights into the cognitive domains that show potential response to treatment targeting the cholinergic system and suggests that future studies would benefit from a more comprehensive evaluation of domain-specific cognitive functions, including protocols for the assessment of attention and executive dysfunction, to further elucidate which cortical networks may benefit from long-term treatment with second-generation ChEIs in patients with mild to moderate AD.

## Abbreviations

AD: Alzheimer disease; ChEI: cholinesterase inhibitor; DRS: Dementia Rating Scale; MMSE: Mini-Mental State Examination.

## Competing interests

This study was conducted independently of any pharmaceutical company sponsorship. SEB has received honoraria for CME and *ad hoc* consulting from Pfizer, Janssen-Ortho, Novartis Pharmaceuticals, Lundbeck, Eisai, GlaxoSmithKline, Schering-Plough, Bristol-Myers Squibb, Elan, and Wyeth Pharmaceuticals, and operating funds from Pfizer Inc., Novartis Pharmaceuticals, Myriad Pharmaceuticals, Roche, and GlaxoSmithKline. KLL has received consultant and speaker honoraria and contract research funding from Pfizer Inc., Wyeth, Lundbeck, and Janssen Ortho. The remaining authors have no competing interests.

## Authors’ contribution

SEB and DTS helped with the writing and conceptual framework of the manuscript. KLL provided feedback on the manuscript. DLS, AK, and JDE provided statistical expertise for the manuscript. JDE also helped with the writing of Methods and Results. PB helped with the conceptual framework, analyses, and writing of the introduction, methods, results, discussion and conclusion. All authors approved and read the final manuscript.

## Author information

SEB and DTS are co-senior authors.
